# Astrocytic modulation of blood brain barrier: perspectives on Parkinson’s disease

**DOI:** 10.3389/fncel.2014.00211

**Published:** 2014-08-04

**Authors:** Ricardo Cabezas, Marcos Ávila, Janneth Gonzalez, Ramon Santos El-Bachá, Eliana Báez, Luis Miguel García-Segura, Juan Camilo Jurado Coronel, Francisco Capani, Gloria Patricia Cardona-Gomez, George E. Barreto

**Affiliations:** ^1^Departamento de Nutrición y Bioquímica, Facultad de Ciencias, Pontificia Universidad JaverianaBogotá, D.C., Colombia; ^2^Departamento de Biofunção, Universidade Federal da BahiaSalvador, Brazil; ^3^Instituto Cajal, CSICMadrid, Spain; ^4^Laboratorio de Citoarquitectura y Plasticidad Neuronal, Facultad de Medicina, Instituto de Investigaciones cardiológicas Prof. Dr. Alberto C. Taquini (ININCA), UBA-CONICET, Buenos AiresArgentina; ^5^Cellular and Molecular Neurobiology Area, Group of Neuroscience of Antioquia, Faculty of Medicine, SIU, University of Antioquia UdeAMedellín, Colombia

**Keywords:** BBB, astrocytes, reactive astrogliosis, endothelial cells, Parkinson disease

## Abstract

The blood–brain barrier (BBB) is a tightly regulated interface in the Central Nervous System (CNS) that regulates the exchange of molecules in and out from the brain thus maintaining the CNS homeostasis. It is mainly composed of endothelial cells (ECs), pericytes and astrocytes that create a neurovascular unit (NVU) with the adjacent neurons. Astrocytes are essential for the formation and maintenance of the BBB by providing secreted factors that lead to the adequate association between the cells of the BBB and the formation of strong tight junctions. Under neurological disorders, such as chronic cerebral ischemia, brain trauma, Epilepsy, Alzheimer and Parkinson’s Diseases, a disruption of the BBB takes place, involving a lost in the permeability of the barrier and phenotypical changes in both the ECs and astrocytes. In this aspect, it has been established that the process of reactive gliosis is a common feature of astrocytes during BBB disruption, which has a detrimental effect on the barrier function and a subsequent damage in neuronal survival. In this review we discuss the implications of astrocyte functions in the protection of the BBB, and in the development of Parkinson’s disease (PD) and related disorders. Additionally, we highlight the current and future strategies in astrocyte protection aimed at the development of restorative therapies for the BBB in pathological conditions.

## Introduction

The Blood Brain Barrier (BBB) is an essential regulatory component of the neural interface with the brain vasculature. It exerts a tightly regulation in the movement of ions, molecules and cells between the neural cells and the blood (Daneman, 2012; Wong et al., 2013), thus maintaining the ionic homeostasis, hormonal and transmitter levels and transport of nutrients in the brain (Luissint et al., 2012). In this aspect, BBB is important for the separation of neurotransmitters pools and neuroactive agents that regulate brain microenvironment (Abbott et al., 2006). Furthermore, the BBB supplies the brain with different nutrients, exerts a restriction of ionic substances between the blood and the brain through specific ion transporters, regulates the ISF (interstitial fluid), prevents the formation of additional injuries during diseases and cerebrovascular accidents and is an important barrier for the brain transport and metabolization of drugs (Abbott et al., 2006; Daneman, 2012; Wong et al., 2013).

The BBB is composed by brain capillary endothelial cells (ECs), with a specific phenotype located in a strong association with astrocytic endfeet processes and mesenchymal-like cells pericytes. Importantly, the BBB is characterized by the presence of tight junctions between ECs, and the expression of specific polarized transport systems (Luissint et al., 2012). On the other hand, astrocytes through their endfeet establish the link between the endothelial blood flux and neurons, and are important regulators in the formation and maintenance of the BBB (Alvarez et al., 2013). BBB dysfunction has been associated with pathological conditions and diseases including cerebral ischemia, brain trauma, glioblastoma, stroke, multiple sclerosis, epilepsy, Alzheimer and Parkinson’s Disease (PD; Haseloff et al., 2005; Daneman, 2012; Alvarez et al., 2013).

PD is a progressive neurodegenerative disorder caused by neuronal death in substantia nigra (SN), degeneration of dopaminergic neurotransmission, and the presence of α-synuclein and protein inclusions in neuronal cells, also known as Lewy bodies (Nutt and Wooten, 2005; Halliday and Stevens, 2011). Main symptoms of Parkinson include asymmetrical bradikinesia, rigidity, resting tremor and postural instability (Fernandez, 2012; Singer, 2012). Initiation and progression of PD is dependent upon cellular events, such as failures in the protein degradation machinery, oxidative stress, mitochondrial dysfunction, defects in mitochondrial autophagy (mitophagy) and the continuous accumulation of α-synuclein, driven through cell to cell interactions between glial cells and neurons that ultimately lead to apoptosis (Jenner, 2003; Halliday and Stevens, 2011; Vives-Bauza and Przedborski, 2011). Although there is not a cure for the disease, the most used and cheaper treatment for PD continues to be Levodopa, frequently accompanied by carbidopa or benserazide (Singer, 2012; Ossig and Reichmann, 2013). However, about 40% of patients developed motor fluctuations and dyskinesias after 4 to 6 years of treatment (Ogawa et al., 2005; Fernandez, 2012), demonstrating that further pharmacological research is needed in order to counterbalance these side effects.

Current research suggests that the exact cause of PD remains unknown (Hirsch et al., 2003; Fernandez, 2012; Schwartz and Sabetay, 2012). Mutations in various proteins such as LRRK2, PARK2), phosphatase and tensin homolog (PTEN)-induced putative kinase 1 (PINK1), and (DJ-1) have been observed in familiar cases of Parkinson, which only account for 10–15% of diagnosed cases (Hirsch et al., 2003; Rappold and Tieu, 2010; Pan-Montojo et al., 2010; Wang et al., 2011). Similarly, various environmental factors have been found to induce PD-like symptoms, including vascular insults to the brain, oxidative stress, neuroleptic drugs, heavy metals exposure and the exposure to pesticides like rotenone or paraquat (Betarbet et al., 2000; Brown et al., 2006; Rappold and Tieu, 2010; Tanner et al., 2011). Similarly, there is clinical and *in vitro* evidence of BBB disruption during PD development (Kortekaas et al., 2005; Hirano et al., 2008; Ohlin et al., 2011; Lee and Pienaar, 2014). In this aspect, previous studies have suggested that α-synuclein deposition has an increase in BBB permeability (Jangula and Murphy, 2013), suggesting the importance of α-synuclein in BBB disruption and PD development. (Braak et al., 2006; Halliday and Stevens, 2011).

A great body of research has shown the importance of astrocytes in the maintenance of BBB properties both during normal and pathological conditions (Ramaswamy and Kordower, 2009; Yasuda and Mochizuki, 2010; Alvarez et al., 2013). Astrocytic secreted molecules are important for the regulation of interactions between BBB components such as ECs and pericytes (Alvarez et al., 2013; Lee and Pienaar, 2014). Furthermore, astrocytes produce antioxidative molecules like GSH, ascorbate and SOD (superoxide dismutase) and a great number of growth factors and neurotrophins, important for brain cell survival during neurodegenerative processes (Dringen, 2000; Ramaswamy and Kordower, 2009; Yasuda and Mochizuki, 2010; Zheng et al., 2010; Barreto et al., 2011).

In the present review we provide a throughout overview of the astrocytic functions in the BBB and its importance during pathophysiological events elicited in PD. Additionally, we highlight the current and future strategies in astrocyte protection aimed at the development of restorative therapies for the BBB in pathological conditions.

## Components of the BBB

### Endothelial cells

ECs within the brain have a characteristic phenotype that makes them different from EC located elsewhere (Dejana, 2004; Stamatovic et al., 2008; Nag, 2011; Daneman, 2012). For example, brain ECs have similarities with epithelial cells, as they are polarized cells that express some specific transporters and in that they are connected by circumferential tight junctions that interfere with the paracellular transport of molecules and ions between cells (Nag, 2011; Daneman, 2012). As well, brain EC have an increased density of mitochondria when compared with the peripheral vasculature, suggesting a higher risk of reactive oxygen species (ROS) formation (Nag, 2011; Lee and Pienaar, 2014). Structurally, EC are in contact with astrocytic endfeet and pericyte through the basal lamina, thus forming the neurovascular unit (NVU), with neurons (Hawkins and Davis, 2005; Stanimirovic and Friedman, 2012; Najjar et al., 2013).

Among its functions in BBB maintenance, EC are important in the bidirectional transport across the brain through ion transporters, protein and peptide carriers and active efflux transport (Nag, 2011). Furthermore, EC have highly organized tight and adherent junctions which restrict the passage of polar substances including hexose sugars, amino acids, nucleosides monocarboxylic acids, and vitamins (Grammas et al., 2011; Mokgokong et al., 2014). Importantly, the integrity of tight junctions is essential to prevent the paracellular transport of many molecules and ions, and its disruption is associated with pathological events in the brain such as microbial infection, cancer, inflammatory responses, stroke, Alzheimer disease and PD (Stamatovic et al., 2008; Luissint et al., 2012). Moreover, some studies have shown alterations in endothelial tight junctions during PD development (Kim et al., 2003; Chen et al., 2008; Lee and Pienaar, 2014). For example, Chen et al. (2008) found a decrease in the tight junction proteins occludin and ZO-1 in a MPTP murine model of PD. Similarly, the exposure of murine EC to ROS increased the activity of metalloproteinase-9 (MMP-9), which caused degradation of the basal lamina and BBB disruption. This oxidative damage was reduced by the overexpression of SOD1 and catalase, suggesting the importance of oxidative stress in BBB disruption (Kim et al., 2003). Additionally, there is *in vitro* and clinical evidence of angiogenic activity in PD development caused by an upregulation in the expression of vascular endothelial growth factor (VEGF; Wada et al., 2006; Lee and Pienaar, 2014). In summary, the cellular and molecular properties of brain ECs are essential for maintaining BBB permeability through an adequate ionic balance, conservation of the junctional structure and an adequate interaction with cells of the NVU.

### Pericytes

Pericytes are enwrapping cells of blood microvessels, and are located between the EC and astrocytic endfeet and neurons (Wong et al., 2013). They are important regulatory cells for the maintenance of both homeostasis and hemostasis in the BBB (Dore-Duffy and Cleary, 2011). Additionally, pericytes are relevant in functions such as stromal regeneration, angiogenesis and neovascularization, antigen presenting cells under brain pathologies, control of EC proliferation, and promotion of neural stem cell properties (Lange et al., 2013; Elali et al., 2014; Hurtado-Alvarado et al., 2014). In this regard, pericytes have shown to differentiate *in vitro* into chondrocytes, vascular smooth muscles cells (VsMCS), osteoblasts and skeletal muscle, suggesting a promising clinical use for pericytes in Central Nervous System (CNS) injuries and other pathologies (Armulik et al., 2010; Lange et al., 2013). Both pericytes and EC are enveloped by a basal membrane that is continuous between the two cell types, which separates pericytes from astrocyte endfeet (Sá-Pereira et al., 2012). This association is achieved through the endothelial secretion of PDGF-B and other angiogenic factors such as VEGF, TGF-β and angiopoietins (Angs), through the interaction of multiple signaling pathways (Dore-Duffy, 2008; Armulik et al., 2010; Ribatti et al., 2011).

Morphologically, pericytes exhibit an oval cell body with a great number of projections that enwrap ECs in different patterns, along the abluminal surface (Armulik et al., 2010). The two main types of pericytes, granular (95% of total pericytes) and agranular have been described in the brain according to the presence or absence of lysosome granules in the cytoplasm. Interestingly, alterations in granular pericytes have been associated with amyloid deposition, and lipid accumulation in human brain cultures, suggesting the importance of pericyte alterations in Alzheimer disease and other pathologies (Castejón, 2011).

Of greater importance are the interactions between astrocytes and pericytes. In this aspect, it has been shown that both pericytes and astrocytes are essential for brain vasculogenesis and BBB maintenance possibly through the activation of PDGFRB signaling (Dejana, 2004; Bonkowski et al., 2011). Moreover, both pericytes and astrocytes are important in the preservation of EC tight junctions through the regulation of proteins like occludin, claudin and ZO-1 (zona occludens-1, Haseloff et al., 2005; Wolburg et al., 2009; Bonkowski et al., 2011). This result suggests the importance of astrocyte-pericyte communication in brain physiology. However, further research is needed in order to understand the implications of the mentioned interactions during neurodegenerative disorders.

### Astrocytes

Astrocytes are the most common cell type in the mammalian brain, conforming the glia with oligodendrocytes and microglia (Chen and Swanson, 2003). Among its many functions, astrocytes are essential for many metabolic processes in the brain such as the promotion of neurovascular coupling, the attraction of cells through the release of chemokines, K^+^ buffering, release of gliotransmitters, release of glutamate by calcium signaling, control of brain pH, metabolization of dopamine and other substrates by monoamine oxidases, uptake of glutamate and γ-aminobutyricacid (GABA) by specific transporters and production of antioxidant compounds like glutathione (GSH) and enzymes such as superoxide dismutases (SODs; Volterra and Meldolesi, 2005; Chinta and Andersen, 2008; Hamby and Sofroniew, 2010; Kimelberg and Nedergaard, 2010; Parpura et al., 2011).

Globally, astrocytes are characterized by the expression of the intermediate filaments vimentin (Vim) and glial fibrillary acidic protein (GFAP), which are upregulated under CNS insults, in a process known as astrogliosis (Volterra and Meldolesi, 2005; Hamby and Sofroniew, 2010; Céspedes et al., 2013). Morphologically, astrocytes are characterized by a stellate shape with multiple processes and ramifications (Chen and Swanson, 2003; Volterra and Meldolesi, 2005), and become activated following brain injuries and degenerative diseases (Barreto et al., 2007, 2009, 2011, 2012; Adelson et al., 2012).

Although a great heterogeneity exists among astrocytes, two main types have been described in the CNS: protoplasmic astrocytes of the grey matter which envelope neuronal bodies and synapses, and fibrous astrocytes from the white matter that interact with the nodes of Ranvier and oligodendroglia (Halliday and Stevens, 2011; Oberheim et al., 2012). Current research has suggested that only protoplasmic astrocytes have an increase in the accumulation of α-synuclein, and these are of importance for PD development (Braak et al., 2006; Halliday and Stevens, 2011). Interestingly, protoplasmic astrocytes are arranged in non-overlapping domains forming a syncytial network that may contact approximately 160.000 synapses, thus integrating neural activity with the vascular network (Bushong et al., 2002; Barreto et al., 2011). This architecture is altered under pathological events such as Alzheimer and Epilepsia and has been associated with reactive astrogliosis (Oberheim et al., 2012), suggesting the importance of structural alterations during damaging processes.

Astrocytic terminal processes, known as endfeet, contact the brain vasculature surface facing ECs and pericytes and enwrap the neuronal synapses, enabling the modulation of both neuronal activity and cerebral blood flow, following an elevation in intracellular Ca^2+^ levels in the endfeet (Zonta et al., 2003; Maragakis and Rothstein, 2006). Importantly, astrocytic endfeet express specialized molecules such as Kir4.1 K^+^ channels and aquaporin 4 that regulate BBB ionic concentrations, and protein transporters such as glucose transporter-1 and P-glycoprotein, suggesting the importance of the endfeet in astrocyte polarization (Abbott et al., 2006; Nag, 2011). Additionally, astrocytes communicate between each other through gap junctions forming a functional syncitium with well-coordinated responses (Theis et al., 2005; Alvarez et al., 2013). In this aspect, it has been suggested that the astrocytic mechanisms that regulate vasodilation and vasoconstriction are transmitted through this inter-astroglial gap junctions (Alvarez et al., 2013). Furthermore, astrocytes are important in the development and maintenance of BBB characteristics in ECs through the release of growth factors like VEGF, glial cell line-derived neurotrophic factor (GDNF), basic fibroblast growth factor (bFGF), and ANG-1 (Alvarez et al., 2013; Wong et al., 2013). These growth factors are important in the formation of tight junctions, the promotion of enzymatic systems and the polarization of transporters (Wong et al., 2013). Astrocyte-secreted growth factors are also important for neuronal growth and maintenance, and have survival properties during brain damaging processes like PD (Hamby and Sofroniew, 2010).

### Extracellular Matrix (ECM)

In addition to the different cell types which constitute the BBB, the extracellular Matrix (ECM) is an important structural element of the BBB that serves as an anchor for the endothelium through the interaction of endothelial integrin receptors and matrix proteins such as laminin (Hawkins and Davis, 2005). In the brain, the ECM is composed of hyaluronan, hyaluronic acid, lecticans, proteoglicans and tenascins, which are important for the maintenance of the paracellular diffusion in the BBB (Hawkins and Davis, 2005; Wong et al., 2013). Previous studies have suggested that the disruption of the ECM is strongly associated with an increase in BBB permeability during pathogenic states such as glioblastoma multiforme, ischemia and hemorrhagic necrosis of the brain. For example, during ischemia, the basement membrane suffers a breakdown caused by the increased expression of the matrix metalloproteinases (MMPs) MMP9 and MMP2 which in addition may cause microglial activation (del Zoppo and Milner, 2006; Lau et al., 2013). Furthermore, increased expression of MMP9 and GFAP in astrocytes was observed in a parkinsonian mouse model with MPTP (1-methyl-4-phenyl-1,2,3,6-tetrahydropyridine; Annese et al., 2014). These results suggest the importance of ECM breakdown in glial activation during neurodegeneration and PD.

## Parkinson disease and BBB

### Causes of disruption of the BBB

Several processes may affect the integrity of the BBB, including an increase in ROS production, elevated levels of proinflammatory cytokines, inappropriate clearance of Ab peptide and other toxic substances (Minagar and Alexander, 2003; Popescu et al., 2009; van Sorge and Doran, 2012). Previous studies have shown an increase in BBB permeability associated with age that is in part responsible for pathological alterations such as white matter lesions (Simpson et al., 2007; Popescu et al., 2009). In this aspect, it has been reported that elder individuals and senescence mouse models have a higher albumin and IgG concentration than younger individuals caused by a leakage through the BBB (Popescu et al., 2009). Moreover, ageing is also associated with an increased production of ROS and proinflammatory cytokines in vascular ECs, which have been linked with memory and learning impairment in mouse models (Fukui et al., 2001; Popescu et al., 2009; Enciu and Popescu, 2013). Aged people have shown a diminished activity of the P-glycopotein efflux transporter that is associated with a limited removal of toxic substances from the brain (Popescu et al., 2009), demonstrating an important correlation between ageing processes (such as the increased expression of ROS) and BBB dysfunction. Taking into account that PD is associated with both age and ROS production, it is important to explore the cellular and molecular mechanisms that are activated during BBB disruption in this pathology and its protective mechanisms.

### Disruption of BBB in Parkinson disease

**Figure 1:**
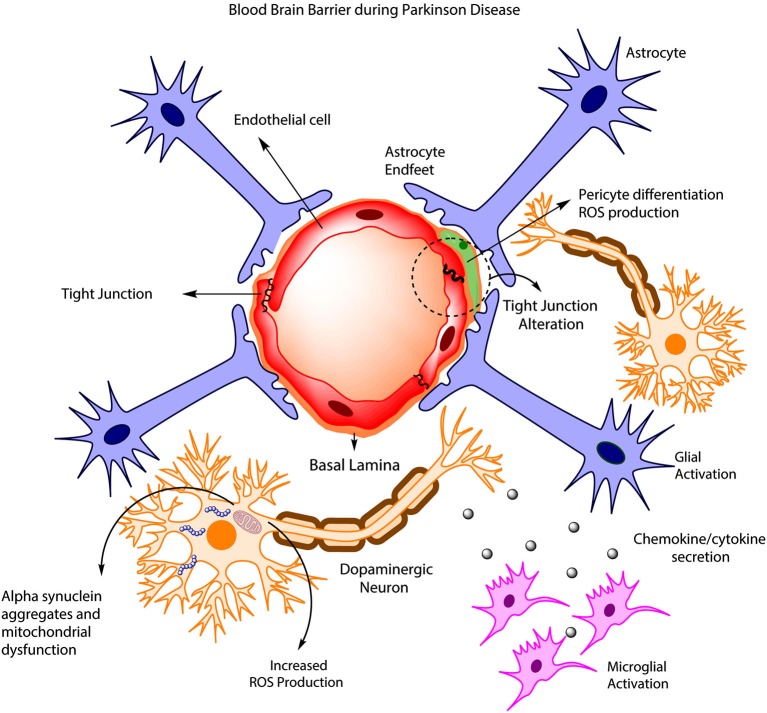
**BBB disruption in PD**. During PD development, increased ROS production leads to the accumulation of α-synuclein in DAneurons, and this is accompanied by mitochondrial dysfunction and increased neuronal death. Concurrently, astrocyte and microglia became activated, promoting cytokine release, which in turn affects endothelial tight junctions, pericyte phenotype and BBB permeability.

Disruption of BBB in PD has been quite controversial. It was initially assumed that BBB remained unaltered during the development of the pathology, as observed in animal models and permeability studies of PD drugs such as levodopa and benserazide (Kurkowska-Jastrzebska et al., 1999; Haussermann et al., 2001). More recently, clinical studies have presented evidence of BBB disruption in PD patients (Kortekaas et al., 2005; Hirano et al., 2008; Ohlin et al., 2011; Lee and Pienaar, 2014). For example, an early study (Kortekaas et al., 2005) pointed out an increase in the brain uptake of drugs that usually do not cross the BBB including benzerazide and [^11^C] verapamil in PD patients and rat models, suggesting a possible BBB breakdown. Additionally, a PET study (positron emission tomography) found deficiencies in cerebral blow flow in PD patients that were highly associated with dyskinesias and levodopa treatment (Hirano et al., 2008). These changes in cerebral blood flow have been associated with an increased BBB permeability and angiogenesis that are mediated by VEGF (Kortekaas et al., 2005; Ohlin et al., 2011). Similarly, various toxin-induced PD models have shown BBB disruption, including 6-OHDA treated rats and MPTP-treated mice (Carvey et al., 2005; Chen et al., 2008). On the other hand, a growing body of evidence has shown the importance of ABC multidrug transporters such as P-gp in BBB disruption (Kortekaas et al., 2005; Bartels et al., 2008; Bartels, 2011). In this aspect, KO mice for P-glycoprotein have shown an increased accumulation of neurotoxin ivermectin and the carcinostatic drug vinblanstine in the brain, suggesting the importance of P-glycoprotein in the clearance of toxic substances and a possible BBB disruption in PD (Schinkel et al., 1994). Additionally, Kortekaas et al. (2005) has suggested that Parkinson patients have a reduced P-gp (glycoprotein) function in the midbrain, which is associated with a BBB disruption. Interestingly, some PET studies reported a decrease in BBB P-gp function in several brain regions during aging, demonstrating that elder people are more susceptible to the accumulation of toxin compounds in the brain. Taking into account that α-synuclein accumulation is associated with PD pathogenesis, it is possible that a reduction of P-gp could be related with an accumulation of α-synuclein in the brain (Bartels, 2011). However, further research is needed to assess the importance of P-gp in this process. Finally, the release of proinflammatory cytokines by microglia and astrocytes during PD is associated with both an increased neuronal death and protein rearrangements in tight junctions on EC surface (Figure 1; Desai Bradaric et al., 2012). For example, increased levels of the cytokines IL-6, IL-1B and TNF-A and a decrease in proteins ZO-1 and occludin in tight junctions have been associated with a reduction in the trans-endothelial electrical resistance, suggesting an alteration in BBB permeability (Wong et al., 2004). Importantly, the loss of signaling interactions between astrocytes and CNS vasculature through changes in protein expression in astrocytic endfeet is associated with morphological changes including hypertrophy, upregulation of GFAP and vimentin and therefore triggering the induction of astrocytes to a more reactive state (Robel et al., 2009; Alvarez et al., 2013). These results highlight the importance of astrocytes in the modulation of BBB properties and the involvement of the reactive astrogliosis during BBB disruption (Figure 1).

### Reactive astrogliosis in PD

Reactive astrogliosis is the main reaction of astrocytes following brain insults such as infection, inflammatory processes, trauma, α-synuclein accumulation, ischemia and neurodegenerative diseases (Barreto et al., 2007, 2009; Gu et al., 2010; Hamby and Sofroniew, 2010; Xiong et al., 2011; Adelson et al., 2012). This process involves both molecular and morphological changes in astrocytes, which include the hypertrophy of cell bodies and glial processes, increased expression of proteins like GFAP, vimentin, nestin, tenascin-C and chondroitin sulfate proteoglycans (CSPGs; Alvarez et al., 2013). Other characteristics of the process are the increased uptake of glutamate caused by an alteration of vesicular transporters of GABA (vGAT) and glutamate (vGLUT), production of cytokines and chemokines that have a modulatory effect on microglia (Croisier and Graeber, 2006), and in some cases the formation of glial scar (Hirsch et al., 2003; Hamby and Sofroniew, 2010; Kang and Hebert, 2011; Colangelo et al., 2014).

Importantly, reactive astrogliosis is a mechanism highly dependent on the cellular and molecular context of the events triggering it, therefore it may have both beneficial and detrimental effects on surrounding neural and non-neural cells (Hamby and Sofroniew, 2010). For example, the glial scar produced after severe astrogliosis may separate necrotic tissue from healthy one, but also has the detrimental effect of impairing axonal regeneration through the expression of molecules like CSPGs, semaphorins and ephrin (Fitch and Silver, 2008; Duffy et al., 2009).

**Figure 2:**
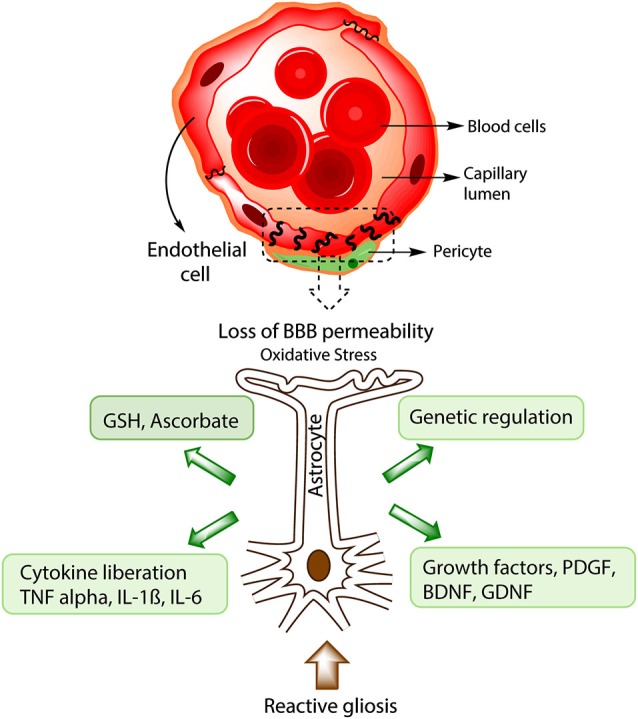
**Protective strategies of astrocytes during BBB disruption**. In advanced stages of PD, BBB disruption takes place and causes a lost in barrier permeability, entrance of toxic substances and in some instances immune cell infiltration. Processes such as increased ROS production, reactive gliosis and cellular death will inevitably occur. Astrocytic response to BBB disruption includes the production of antioxidative molecules like GSH and ascorbate, generation of growth factors like Brain derived neurotrophic factor (BDNF) and GDNF that could alleviate the cellular death and promote angiogenesis. Furthermore, astrocytes are important in the genetic regulation of endothelial proteins from the tight junction like Occludin and ZO-1. During chronic brain damage, astrocytes also induce the liberation of cytokines like TNF-α, IL-1B, IL-6, important in microglial activation and neuronal death.

Experimental evidence using cellular and animal models have shown that environmental and biological toxins, like α-synuclein, LPS (lipopolysaccharides), herbicides and pesticides like rotenone or MPTP (1-methyl-4-phenyl-1,2,3,6-tetrahydropyridine), can induce both astrogliosis and microgliosis, which is accompanied by altered striatal neuronal morphology, neuronal death, mitochondrial dysfunction and nuclear fragmentation (Langston et al., 1999; Samantaray et al., 2007; Niranjan et al., 2010). Additionally, injection of LPS in rat brains was followed by an increase in the inducible nitric oxide synthase (iNOS), suggesting that chronic glial activation can cause oxidative stress in the brain, similarly to that in neurodegenerative processes like AD and Parkinson (Sugaya et al., 1998; Hirsch et al., 2003; de Oliveira et al., 2011). Similarly, there is clinical evidence showing that astrogliosis is present in different areas of the brain in PD patients, including the SN, the putamen and the hippocampus (Baxendale et al., 1998; Dickson et al., 2002; Dickson, 2012). Finally, some studies have shown that activated glial cells can participate in the death of dopaminergic neurons, probably via activation of apoptosis by cytokines like TNF-α, IL-1B, IL-6 (Figure 2) and interferon-γ and the subsequent production of nitric oxide by the iNOS that may diffuse toward the neurons and induce lipid peroxidation, DNA strands breaks and inhibition of mitochondrial metabolism (Hirsch et al., 2003; Rappold and Tieu, 2010). Released cytokines may bind to TNFR1 and 2, specific receptors in dopaminergic neurons, and activate proapoptotic mechanisms through the activation of caspase 3, caspase 8, and cytochrome C (Hirsch et al., 2003). These results suggest that both the glial reaction and the consequent inflammatory processes could be considered as a promising therapy to reduce neuronal damage during PD (Hirsch et al., 2003).

## Protection strategies of astrocytes in BBB disruption

Over the last years, much research has focused on specific molecules produced by astrocytes as promising neuroprotective strategies in neuropathologies. These molecules include antioxidant enzymes such as SODs, growth factors, peptide hormones and heat shock proteins (Dringen, 2000; Zheng et al., 2010; Barreto et al., 2011). Many of them have shown protective effects both in dopaminergic neurons and glial cells, and have been used in animal models and clinical trials with remarkable results (Ramaswamy and Kordower, 2009; Yasuda and Mochizuki, 2010). In the last section of our review we discuss the current methods used in neuroprotection based on astrocyte molecules. Additionally, we highlight the future strategies in astrocyte protection aimed at the development of restorative therapies for the BBB in pathological conditions.

### Astrocytic antioxidants and PD

Astrocytes secrete beneficial antioxidant molecules, including GSH, (SODs 1, 2 and 3), and ascorbate, which are important for cell survival during neurodegenerative processes (see Figure 2, Anderson and Swanson, 2000; Dringen, 2000; Lindenau et al., 2000; Sims et al., 2004; Mythri et al., 2011). The tripeptide GSH is the main antioxidant in the brain, which is needed for the conversion of methylglyoxal into d-lactate by glyoxalase 1 (Dringen, 2000; Bambrick et al., 2004). Furthermore, GSH is also important in limiting and repairing the deleterious actions of NO and other ROS in the brain such as nitrations and fibril formations of α-synuclein (Chinta and Andersen, 2008). Interestingly, astrocytes possess a greater concentration of GSH (3.8 mmol/L) than neurons (2.5 mmol) probably due to their higher content of γ-glutamylcysteine-synthetase (Rappold and Tieu, 2010). In this aspect, some studies demonstrated that neurons co-cultured with astrocytes exhibit higher levels of GSH compared to neurons cultured alone, suggesting that astrocytes may provide further antioxidant defenses to neurons and BBB cells (Maier and Chan, 2002; Slemmer et al., 2008; Giordano et al., 2009). Additionally, an increase in GSH peroxidase-containing cells showed to be inversely correlated with the severity of dopaminergic cell loss in cell populations from patients with PD, demonstrating that the quantity of GSH peroxidase in cells might be critical for a protective effect against oxidative stress during PD (Damier et al., 1993). Different murine models have shown the importance of GSH in BBB protection, including maintenance of BBB permeability and oxidative protection of mouse pericytes (Shukla et al., 1993; Agarwal and Shukla, 1999; Price et al., 2012). Similarly, ascorbate was shown to protect BBB integrity in a rat ischemic model by preventing changes in BBB permeability and increased ROS production (Lin et al., 2010). One important problem with the use of GSH as a possible therapeutic agent is that its precursor, N-acetycysteine (NAC), does not cross the BBB in significant amounts, therefore various strategies have been used to improve the GSH transport into the brain, such as the use of liposomes, nanoparticles and L-dopa conjugates (Smeyne and Smeyne, 2013). However, further research is needed to address the use of GSH in BBB disruption.

Previous studies showed that SODs exert neuroprotection in PD and other oxidative-related events (Chen and Swanson, 2003). For example, the overexpression of Cu/Zn SOD (SOD1) was able to rescue dopaminergic neurons and diminishes locomotor disabilities in a *Drosophila* mutant model for α-synuclein overexpression (Botella et al., 2008). Interestingly, a specific increase in SOD levels in the SN, with no changes in activities of GSH peroxidase, catalase and GSH reductase, is observed in PD patients (Chinta and Andersen, 2008). A similar increase was noted in the mitochondrial isoform of SOD (SOD2) in motor cortex from PD patients (Radunović et al., 1997), suggesting that SODs have a greater importance than other antioxidant enzymes during PD development. Furthermore, the reduction or induced mutation of SOD1 in astrocytes has been shown to induce neuronal degeneration and injury in ischemic and amyotrophic lateral sclerosis (ALS) murine models (Kondo et al., 1997; Kim et al., 2001; Blackburn et al., 2009; Papadeas et al., 2011). Finally, it was also reported that the overexpression of SOD1 in a transgenic mouse model attenuated BBB disruption by superoxide anion during ischemia (Kim et al., 2001). Altogether, these results emphasize the importance of antioxidant enzymes for the treatment of PD and BBB disruption.

### Growth factors and BBB protection in PD

Several neurotrophic and growth factors secreted by astrocytes have been extensively used in animal models of neurodegenerative disorders for exerting protection of dopaminergic neurons and glial cells against toxins and ROS during injury through the activation of specific signaling pathways that are responsible for cell survival, induction of antioxidant enzymes, and axonal sprouting (See Figure 2, Ramaswamy and Kordower, 2009; Yasuda and Mochizuki, 2010; Proschel et al., 2014). Some of them like GDNF and neurturin (NRTN) have been tested in clinical trials for PD and other neurodegenerative diseases (Peterson and Nutt, 2008; Ramaswamy and Kordower, 2009).

BDNF, from the neurotrophin family, has been shown to be critical in the survival of cortical, hippocampal and serotonergic neurons. Reduction in BDNF levels is associated with many pathological conditions such as PD, AD, Huntington Disease, ALS, depression and schizophrenia (Allen et al., 2013). Furthermore, BDNF protects neurons against excitotoxicity through activation of the transcription factor NF-kB, which induces expression of antioxidant enzymes such as Mn-SOD and the anti-apoptotic proteins, Bcl-2 and inhibitor of apoptosis proteins IAPs (Mattson, 2008; Lee et al., 2009). Endogenous administration of BDNF was demonstrated to protect neurons in SN following 6-OHDA and MPTP toxicity in rat and primate PD models (Ramaswamy and Kordower, 2009).

The family of GDNF comprises ligands, such as GDNF, NRTN, artemin (ARTN) and persephin. GDNF, secreted by astrocytes and pericytes, is essential for the survival of dopaminergic neurons, peripheral motor neurons and neurons from the locus coeruleus (Yasuda and Mochizuki, 2010; Allen et al., 2013). In this aspect, GDNF administration by catheter increases dopaminergic neuronal resistance against 6-OHDA toxicity, with preservation of motor functions in rat and rhesus monkey models (Safi et al., 2012). More recently, GDNF was shown to increase the expression of claudin-5 and the transendothelial electrical resistances of brain microvascular ECs, suggesting that it may improve the barrier function of the BBB (Sano et al., 2007). However, clinical trials in patients that were administered GDNF in different regions of the brain have shown mixed results, in part due to the mechanism of administration, and the growth factor inability to cross the BBB, therefore further research is needed in order to surpass this obstacle (Gill et al., 2003; Ramaswamy and Kordower, 2009; Allen et al., 2013).

The family of the fibroblast growth factors (FGF) includes 22 structurally related signaling molecules in humans, such as acid FGF, and bFGF, which are important in processes like angiogenesis, wound healing and embryonic development (Itoh and Ornitz, 2011; Huang et al., 2012). Different studies have shown that bFGF protects hippocampal and cortical neurons against glutamate toxicity by changing the expression of *N*-methyl-D-aspartic acid (NMDA) receptors and antioxidant enzymes like SODs and GSH reductase (Timmer et al., 2004; Mattson, 2008). Furthermore, a co-culture of transgenic Schwann cells overexpressing FGF-2 with dopaminergic neurons improved neuronal survival and the behavioral outcome in a parkinsonian rat model lesioned with 6-OHDA (Timmer et al., 2004). Additionally, bFGF preserves BBB endothelial adherens junctions in a mouse model of intracerebral hemorrhage through the inhibition of RhoA protein, suggesting that bFGF maintains BBB integrity (Huang et al., 2012). Finally, there are other neurotrophic factors with potential effects on BBB protection including insulin-like growth factors (IGFs), vascular endothelial growth factor (VEGF-B), hepatocyte growth factor (HGF), mesencephalic astrocyte-derived neurotrophic factor and platelet derived growth factor (PDGF; Aberg et al., 2006; Ramaswamy and Kordower, 2009; Pang et al., 2010; Yasuda and Mochizuki, 2010; Sullivan and Toulouse, 2011). For instance, VEGF has shown to improve cerebral blood flow and the pericyte coverage of brain ECs in a murine ischemic model (Zechariah et al., 2013). Also, PDGF-BB impairment in mice has been associated with a reduced number of pericytes, edema formation and murine embryonic lethality, suggesting its importance in BBB development and maintenance (Bergers and Song, 2005; Bonkowski et al., 2011).

The main obstacle with the use of growth factors as therapeutic agents in neurodegenerative diseases seems to be their inability to cross the BBB thoroughly (Peterson and Nutt, 2008). In this regard, different strategies have been used including injections into the lumbar or ventricular CSF, viral vectors with growth factor genes, the temporal disruption of the BBB with hyperosmotic agent like mannitol, the use of linked peptides or peptidomimetic monoclonal antibodies or nanoparticles (Allen et al., 2013). For example, a recent methodology using magnetic nanocarriers for the transport of BDNF was able to cross the BBB without affecting cell viability seems promising (Pilakka-Kanthikeel et al., 2013). A different approach seems to be the transplantation of dopaminergic neurons or glial precursor cells into the injured regions of the brain, which increases the expression of growth factors like BDNF, GDNF, and IGF (Hauser, 2011; Jankovic and Poewe, 2012; Proschel et al., 2014). In this aspect, a recent study by Proschel et al. (2014) has demonstrated that the transplantation of glial precursor cells in 6-OHDA injured rats causes the recovery of DA neurons of the striatum by an increase in the levels of GSH, GDNF, and BDNF. These results suggest that growth factors are essential in the recovery of BBB injuries and related pathologies.

## Conclusions and future perspectives

Based on the past studies, it seems to be of greater importance to understand the role of BBB in neurodegenerative diseases. It is likely that the maintenance of the BBB and the NVU will decrease the accumulation of Lewy bodies, α-synuclein fibrils and ROS that worsen the effects of PD. It is important to determine the extent of BBB disruption in PD, and how this disruption may allow the transport of growth factors and antioxidant molecules to the site of injury. The combination of novel drug therapies, such as the use of growth factors, antioxidant molecules or nanoparticles combined with a better understanding of the astrocytic functions in the BBB, and the use of other therapies that increase astrocyte survival and its antioxidant function may shed light on a prospective cure of PD in the near future.

## Conflict of interest statement

The authors declare that the research was conducted in the absence of any commercial or financial relationships that could be construed as a potential conflict of interest.
